# Survival Analysis in Patients with Lung Cancer and Subsequent Primary Cancer: A Nationwide Cancer Registry Study

**DOI:** 10.3390/jcm11195944

**Published:** 2022-10-08

**Authors:** Wen-Ru Chou, Ben-Chang Shia, Yen-Chun Huang, Chieh-Wen Ho, Mingchih Chen

**Affiliations:** 1 Department of Internal Medicine, Fu Jen Catholic University Hospital, Fu Jen Catholic University, New Taipei City 242062, Taiwan; 2 Graduate Institute of Business Administration, College of Management, Fu Jen Catholic University, No. 510, Zhongzheng Rd., Xinzhuang Dist., New Taipei City 242062, Taiwan; 3 Artificial Intelligence Development Center, Fu Jen Catholic University, No. 510, Zhongzheng Rd., Xinzhuang Dist., New Taipei City 242062, Taiwan; 4 Department of Life Science, National Taiwan University, Taipei 10617, Taiwan

**Keywords:** lung cancer, multiple primary malignancies (MPMs), survival, epidermal growth factor receptor (EGFR), National Health Insurance Registry Database (NHIRD)

## Abstract

With improved survival in patients with cancer, the risk of developing multiple primary malignancies (MPMs) has increased. We aimed to characterize MPMs involving lung cancer and compare these characteristics between patients with single lung cancer and those with lung cancer and subsequent primary cancer (known as lung cancer first [LCF]). Methods: This retrospective study was conducted based on Taiwan Cancer Database from Taiwan’s National Health Insurance Registry Database. Patients with lung cancer (*n* = 72,219) from 1 January 2011 to 31 December 2015, were included in this study, and their medical records were traced back to 1 January 2002, and followed until 31 December 2019. Results: MPMs occurred in 10,577 (14.65%) patients with lung cancer, and LCF and other cancer first (OCF) accounted for 35.55% and 64.45% of these patients, with a mean age at lung cancer diagnosis of 65.18 and 68.92 years, respectively. The median interval between primary malignancies in the OCF group was significantly longer than that in the LCF group (3.26 vs. 0.11 years, *p* < 0.001). Patients in the single lung cancer group were significantly older than those in the LCF group (67.12 vs. 65.18 years, *p* < 0.001). The mean survival time of patients with LCF was longer than that of patients with single lung cancer. Following initial lung cancer, the three most common second primary malignancies were lung, colon, and breast cancers. For patients with advanced lung cancer, survival in patients with mutant epidermal growth factor receptor (EGFR) was longer than that in patients with undetected EGFR. In stage 3 and 4 patients with EGFR mutations, the LCF group showed better survival than the single lung cancer group. Conversely, in stage 1 patients with mutant EGFR, the LCF group exhibited worse survival than the single lung cancer group. Conclusions: Survival in patients with MPMs depends on baseline characteristics and treatments. Our findings may contribute to the development of precision medicine for improving personalized treatment and survival as well as the reduction of medical costs.

## 1. Introduction

In Taiwan, lung cancer is the most common cause of cancer deaths, resulting in over 9000 deaths per year [1]. Lung cancer is also the top cause of cancer deaths in the United States, causing approximately 28% of cancer deaths [2,3]. According to data from the Surveillance, Epidemiology and End Results database, the incidence of lung cancer increased and peaked in 1992. The survival rate of lung cancer has increased in the past four decades [2,4]. The average 10-year survival rate in patients with early-stage tumors is 88%, which may increase to 92% if they undergo surgical resection immediately after diagnosis [5]. However, improved survival in patients with cancer increases the risk of developing subsequent primary malignancies. In the United States, one in five newly diagnosed patients with cancer has a history of cancer. The subsequent primary cancers significantly impact morbidity and mortality in cancer survivors [6]. Previous reports have revealed that the incidence of multiple primary malignancies (MPMs), defined as at least two independent primary malignancies in the same or different organs of the same individual, has increased [7,8]. The incidence of MPMs has been reported as 0.73–5.2% in all patients with tumors. This large discrepancy may be attributed to different experiences of physicians and different diagnostic tools used by various hospitals [7]. The clinical manifestations and prognostic factors of MPMs involving lung cancer have been addressed in several studies in the past decades [7,9,10,11], but some conclusions are controversial. Numerous studies have demonstrated that patients with MPMs do not have worse prognoses than those with single cancers [9,10]; however, opposite results were obtained by other studies [12]. Independent prognostic factors include the stage of lung cancer, age, smoking, and interval between primary malignancies [11,13,14]. Furthermore, sex and order of MPM occurrences affect the prognosis of patients with MPMs [10,11]. Sex also plays a role in the development of comorbid cancers, including esophageal squamous cell carcinoma and laryngeal cancers [15]. Most published studies are based on single-centers or regional cancer registries [16], and few national database studies have been performed. Additionally, the epidemiologic data varied between different countries or regions, and there is limited information on subgroup analyses of different cancer stages and epidermal growth factor receptor (EGFR) expression. Therefore, in the present study, a thorough analysis using data from multiple hospitals and institutions was performed. This study aimed to investigate the characteristics and prognosis of MPMs involving lung cancer and compare these characteristics between patients with single lung cancer and those with lung cancer and subsequent primary cancer (known as lung cancer first [LCF]). Finally, subgroup analyses were performed to investigate the effects of different cancer stages and EGFR expression on prognosis in patients with single lung cancer and LCF.

## 2. Materials and Methods

### 2.1. Data Sources and Research Samples

Taiwan’s National Health Insurance Registry Database (NHIRD) is a comprehensive real-world database established in 1995. NHIRD provides information on over 99% of individuals in Taiwan, including hospitalization and outpatient attendance [17]. Taiwan Cancer Registry (TCR) files are included in NHIRD and contain detailed information on cancer diagnoses and treatments.

The rate of TCR completeness is 98.4%. In 2002, TCR recorded the International Classification of Diseases for Oncology (third edition, ICD-O-3), cancer staging, diagnosis and recurrence dates, histological cancer types, and detailed treatment information in long-form files. Furthermore, laboratory and clinical data were included in the long-form files since 2011 [18,19]. Informed consent for this study was fully waived because the personal demographic information was anonymized in NHIRD. The Ethics Institutional Review Board of Fu Jen Catholic University in Taiwan reviewed and approved the study protocol (IRB number: C108121).

### 2.2. Study Population and Exclusion Criteria

This retrospective study included data on patients with lung cancer (*n* = 72,219) retrieved from the TCR files from 1 January 2011 to 31 December 2015. The medical records of patients diagnosed with lung cancers were traced back to 1 January 2002, and followed until 31 December 2019, to identify existing or subsequent second primary cancers. The longest follow-up time was 14.7 years. The second primary cancer was identified based on the coding of another primary cancer in the registry in a different organ or with pathology distinguished from that of the primary lung cancer, according to the criteria proposed by Warren and Gates [20]. TCR is recorded with strict clinical judgment of pathological results. Thus, metastatic cancer was recorded as a recurrence of an initial primary cancer and not as a second primary cancer. The date of lung cancer diagnosis (ICD-9-CM: 162/ ICD-O-3: C34) was set as the index date, and the follow-up time was from 1 January 2002 to 31 December 2019. The endpoint was the date of death or end of the follow-up time.

**Figure 1:**
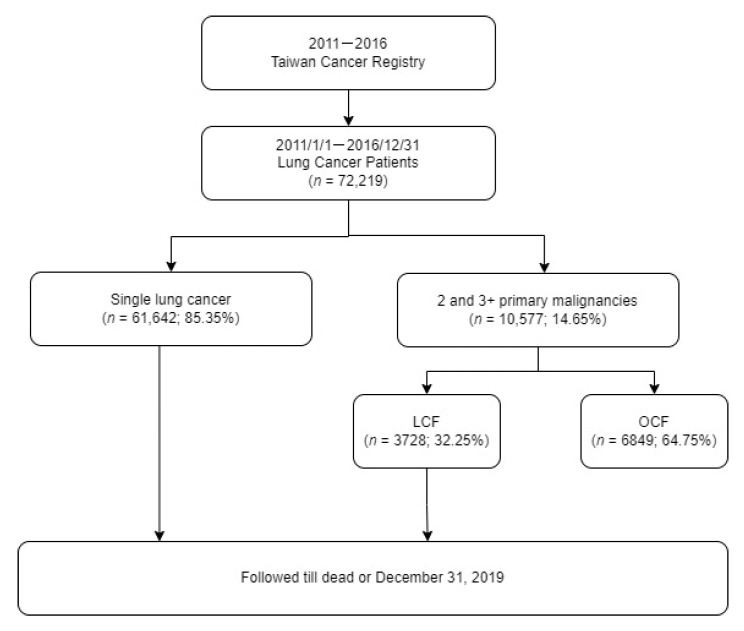
Research flow chart.

Figure 1 presents the research flow chart. Patients with lung cancer were categorized into patients with single lung cancer (*n* = 61,642; 85.35%) and those with two or more malignancies involving lung cancer (*n* = 10,577; 14.65%). Based on the classification proposed by Warren and Gates in 1932, synchronous MPMs (SMPMs) were defined as tumors occurring within ≤6 months of each other, and metachronous MPMs (MMPMs) were defined as primary tumors that developed with a period of >6 months between their occurrences [20]. Further, patients with MPMs involving lung cancer were divided into those with LCF and other cancer first (OCF) based on the order of the primary cancer occurrences. Patients in whom the first malignancy was lung cancer were categorized as those with LCF (*n* = 3728; 32.25%), whereas patients in whom the first malignancy was a cancer other than lung cancer (lung cancer was the second primary cancer) were classified as those with OCFs (*n* = 6849; 64.75%) [9].

### 2.3. Statistical Analyses

Chi-square test was used to compare categorical variables, expressed as N (%), and t-test was used to compare continuous variables, expressed as means ± standard deviations. Multivariate Cox proportional hazard regression analysis with adjusted hazard ratios and 95% confidence intervals for different variables that potentially confounded survival were applied to estimate survival in the single lung cancer and LCF groups, respectively. The Kaplan–Meier method was used to analyze the survival rates of patients with lung cancer. Statistical analyses were performed using SAS 9.4 (SAS Institute, Cary, NC, USA) and R software 3.4.1 version (The Project for Statistical Computing, Vienna, Austria).

## 3. Results

Table 1 summarizes baseline demographic characteristics of 72,219 patients with lung cancer, including 61,642 patients with single cancer and 10,577 patients with two or more primary malignancies. The cumulative incidence of MPMs was 14.65% with a follow-up time of 14.7 years. The LCF group comprised 3728 patients, whereas the OCF group consisted of 6849 patients. Both groups showed a high proportion of male patients over 65 years. The main histological type was adenocarcinoma (AC), which accounted for 65.88% and 60.58% of patients in the LCF and OCF groups, respectively. The mean age at diagnosis of the first primary cancer was 65.18 and 65.28 years, whereas that at the diagnosis of the second primary cancer was 66.03 and 68.92 years in the LCF and OCF groups, respectively. The median interval between the two primary malignancies was 0.11 and 3.26 years in the LCF and OCF groups, respectively. At the time of occurrence, the LCF group had the highest proportion of patients with SMPMs (65.33%). In contrast, most patients had MMPMs (94.48%) in the OCF group. The EGFR mutation rate in the LCF group was significantly higher than that in the OCF group (17.11% vs. 11.02%). A higher percentage of patients underwent surgery in the LCF group than in the OCF group (47.05% vs. 36.75%) (Table 1).

The characteristics of patients with single lung cancer and LCF are shown in Table 2. A higher proportion of patients were over 65 years in the single lung cancer group than in the LCF group (*p* < 0.001). The mean age at diagnosis of lung cancer in the single lung cancer and LCF groups was 67.12 and 65.18 years, respectively (*p* < 0.001). The percentage of patients who were engaged in smoking and alcohol consumption was lower in the LCF group. A higher proportion of patients were diagnosed with stage 4 cancer in the single lung cancer group than in the LCF group (56.06% vs. 29.61%, *p* < 0.001). The distribution of histological type in the single lung cancer group was significantly different from that in the LCF group (*p* < 0.001).

Table 3 and Table S1 show the mean and median survival time. The mean survival time of patients with single lung cancer and LCF are presented in Table 3. The mean survival time of patients with LCF in stages 2, 3, and 4 was longer than that of patients with single lung cancer. In stage 3, the mean survival time of patients with single lung cancer was 2.22 years, whereas that of patients with LCF was 2.96 years. In stage 4, the mean survival time of patients with single lung cancer was 1.35 years, whereas that of patients with LCF was 1.80 years. The mean survival time of patients with a history of smoking, alcohol drinking, and all histological types in the LCF group was longer than that in the single lung cancer group. The mean survival time of patients with EGFR mutations was 2.58 and 3.51 years in the single lung cancer and LCF groups, respectively. The mean survival time of patients without EGFR mutations was 1.77 years in the single lung cancer group and 2.78 years in the LCF group.

Table 4 shows the ten most common second primary cancers in patients with LCF. The three most common cancers after the initial lung cancer were lung (ICD: 162), colon (ICD: 153), and breast (ICD: 174) cancers.

**Figure 2:**
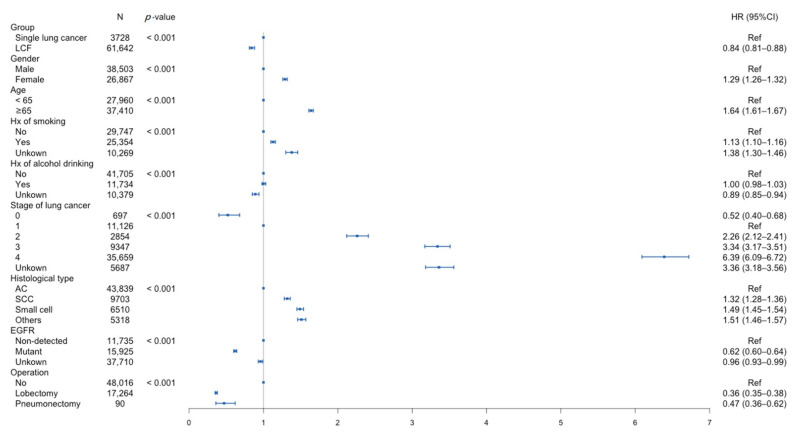
Multivariate analysis of overall survival in patients with single lung cancer and lung cancer first (LCF). AC: adenocarcinoma; EGFR: epidermal growth factor receptor; SCC: squamous cell carcinoma.

Figure 2 and Table S2 present the multivariate analyses of overall survival in the single lung cancer and LCF groups. The LCF group showed significantly worse survival compared with the single lung cancer group, with a hazard ratio of 0.84. Table S2 shows the univariate and multivariate analyses of overall survival in both groups in detail, including crude and adjusted models. The univariate analysis revealed that age, sex, smoking, drinking, EGFR mutation, different cancer stage, histological type, and operation were significantly associated with all-cause mortality.

In Table 5, a matrix plot with pairwise comparisons obtained from the Kaplan–Meier analysis demonstrates the survival in the single lung cancer and LCF groups in different stages with or without EGFR mutations. The survival between single lung cancer and LCF were significantly different at stage 1 and 4 with EGFR mutations (*p* < 0.001).

**Figure 3:**
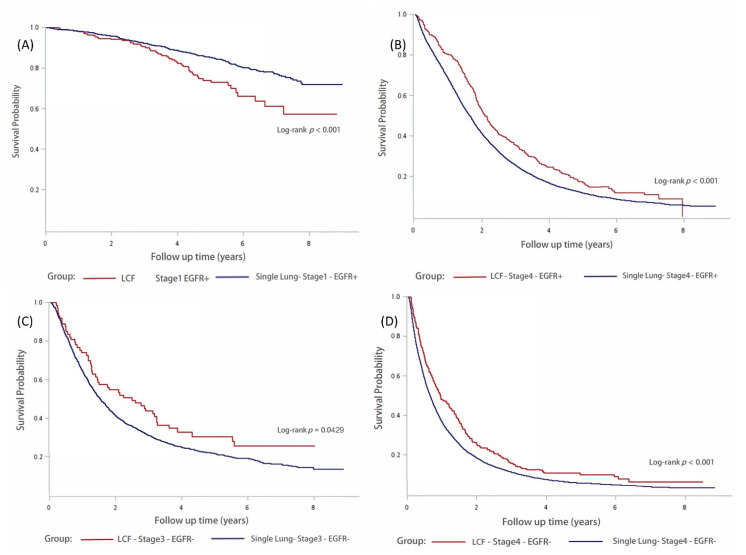
Kaplan–Meier analyses of overall survival between LCF and single lung cancer based on cancer stage and EGFR expression. (**A**) stage 1 patients with EGFR mutations, (**B**) stage 4 patients with EGFR mutations, (**C**) stage 3 patients with undetected EGFR, (**D**) stage 4 patients with undetected EGFR. EGFR: epidermal growth factor receptor; LCF: lung cancer first.

Kaplan–Meier survival curves are shown in Figure 3. In stage 1 patients with EGFR mutations, the single lung cancer group exhibited better survival than the LCF group (Figure 3A). Conversely, in stage 4 patients with EGFR mutations (Figure 3B) or undetected EGFR (Figure 3D), the single lung cancer group showed worse survival than the LCF group. Survival was significantly different between stage 3 (*p* = 0.0429) (Figure 3C) and 4 (*p* < 0.001) (Figure 3D) patients with undetected EGFR.

## 4. Discussion

### 4.1. Heterogeneity in the Incidence of MPMs Involving Lung Cancer

Wide variations in the incidence and characteristics of MPMs involving lung cancer are mainly due to differences in study populations and follow-up time. The characteristics and prognosis of MPMs have been previously reported. MPMs are defined by the diagnostic criteria proposed by Warren and Gates [20], which include the following: “(1) each malignancy must be histologically confirmed, (2) each malignancy occurs in a different region or organ, (3) the new emergent cancer must be confirmed to be non-metastatic, and (4) each cancer has its own pathological features” [21]. MPMs are categorized into two groups based on the timing of the two occurrences. In the LCF group, lung cancer is the first primary malignancy, whereas in the OCF group, lung cancer is the second primary malignancy [9]. However, the definitions of SMPM and MMPM vary in the literature. Although several studies considered 6 months between malignancies as the criteria for MMPM, some studies used a 60-day or 2-year interval as the criteria for distinguishing SMPMs and MMPMs [14,22]. The overall incidence of MPMs varies by country and region, which may be attributed to different study populations, diagnostic techniques, and healthcare system facilities. The risk of developing second primary malignant neoplasm is higher in cancer survivors than in the general population, showing a 3.8% higher probability of developing metachronous second primary malignant neoplasm within a median follow time of 2.5 years [21]. Moreover, the 10-year cumulative risk of developing second primary cancer is as high as 13% if diagnosed in patients aged 60–69 years [21]. The incidence of MPMs is approximately 5% for all tumors [23] and 0.86–6.4% for MPMs involving lung cancer [7,24,25].

Although the duration of follow-up varied in previous studies, the cumulative incidence of MPMs involving lung cancer increased over time. In our study, the cumulative incidence was 14.65% over a follow-up time of 14.7 years. The cumulative incidence in our study was much higher than that reported in previous studies in Asia. A retrospective analysis revealed that 2.5% (364/14,528) of patients with lung cancer developed MPMs over a median follow-up time of 5.37 years [9]. The incidence was potentially underestimated because the follow-up time was relatively short. Another single-center study in Taiwan reported that the incidence of MPMs involving lung cancer during the follow-up was only 0.86%, i.e., 193 of 22,405 patients with cancer had MPMs involving lung cancer between 1993 and 1997 [24]. In the LCF subgroup, the incidence of second primary malignancy also varied widely. In a previous study, the incidence of second primary malignancy following initial primary non-small cell lung cancer (NSCLC) was 6.4%, and lung cancer was the most common second cancer (45.1%) [25]. Similarly, in our study, 3728 (5.16%) of 72,219 patients with lung cancer developed second primary cancers, and the most common second cancer was lung cancer. MPM risk factors have been recently highlighted. A previous study revealed that smoking was a significant risk factor for developing MPMs involving lung cancer [24]. However, the genetic, iatrogenic, or environmental risk factors for MPMs remain unclear [8]. Patients who underwent radiotherapy and chemotherapy had more MPMs than those who did not receive these treatments [26]. Moreover, a study showed that increasing age and being divorced/widowed/separated were independent risk factors for second primary lung cancer (SPLC) in most primary cancer types, and over half of the patients died of SPLC [27]. Men are more likely than women to have second malignant neoplasms [12].

We investigated the clinical characteristics of patients, including a history of smoking, prognosis, and common accompanying malignancies in MPMs involving lung cancer. In our study, 3728 (35.2%) of the 10,577 patients with MPMs involving lung cancer had LCF, and 6849 (64.8%) patients had OCF. These results are consistent with those reported in previous studies [7,16,21,28]. A study in Taiwan showed 26.4% (51/193) had LCF and 73.6% (142/193) had OCF [24]. The mean age at diagnosis of the first and second primary malignancies was significantly different between the LCF and OCF groups [24]. These results were consistent with our results showing that the median interval between the two primary malignancies in the OCF group was 3.26 years, which was significantly longer than that in the LCF group. Moreover, the mean age at diagnosis of lung cancer in the LCF group was lower than that in the single lung cancer group. The most common second primary malignancies accompanying LCF were lung, colon, breast, and prostate cancers in our study. A previous study revealed that upper digestive tract, colorectal, and cervical cancers were the most common cancers accompanying lung cancer [24]. These results varied across sexes and countries. For instance, the most common concomitant malignancies among males with lung cancer were gastric, prostate, and colon cancers, whereas those among females with lung cancer were breast, thyroid, and colon cancers [29]. The incidence of gastric cancer is higher in Japan, and prostate cancer is less frequent in Asian males than in African and Caucasian males [9]. The most common second primary malignancy following NSCLC was lung cancer (45.1%) [25]. Our data consistently showed that the most common second primary malignancy in the LCF group was lung cancer in both males and females.

### 4.2. Survival and Prognosis of MPMs Involving Lung Cancer

In contrast to the conventional belief that patients with multiple cancers exhibit inferior survival rates, a previous study revealed that the survival of patients with second lung cancer coupled with other cancers showed no significant differences compared with that of patients with single lung cancer [10]. Additionally, patients with metachronous MPMs tended to have a better prognosis than those with synchronous MPMs, and the clinical stage was a significant risk factor [9]. Patients with initial lung cancer SMPMs exhibited worse prognosis, and patients with second primary non-lung cancers had better prognosis than those with SPLC [9,30]. We compared survival in the single lung cancer and LCF groups and analyzed the subgroups in different stages and with different EGFR expression. The overall mean survival time of patients with LCF was significantly longer than that of patients with single lung cancer. This difference may be due to survivorship bias or the age at lung cancer diagnosis. Patients with LCF were younger than those with single lung cancer. Univariate analyses revealed that old age, male sex, history of smoking and alcohol drinking, synchronous cancer, small cell lung cancer, late-stage cancer, and undetected EGFR were associated with poor prognosis. Multivariate analyses revealed that old age, male sex, small cell lung cancer, late-stage cancer, and negative EGFR were independent risk factors for poor prognosis. Not surprisingly, these poor prognostic factors for lung cancer were also the prognostic factors for MPMs involving lung cancer.

Asian patients with lung adenocarcinoma are more likely to have EGFR mutations. In China, the overall frequency of EGFR mutations is 50.2%, and the frequency of EGFR mutations in regular smokers is 35.3% [31]. EGFR testing is recommended for all patients with lung cancer in advanced stages (stages 3b, 3c, and 4) of adenocarcinoma, especially in females and nonsmokers [31]. A recent study revealed the impact of EGFR mutations on survival; the clinical stage of lung cancer, order of occurrence of lung cancer, and existence of EGFR mutations were important factors for patient survival [32]. Unlike these studies, we conducted subgroup survival analyses of different stages and EGFR expression statuses. In all stages, the LCF group with or without EGFR mutations had longer mean survival time than the single lung cancer group. Survival differed significantly in different stages and with different EGFR mutation statuses between the LCF and single lung cancer groups. Compared with patients with undetected EGFR, survival in stage 3 and 4 patients with mutant EGFR was superior in both groups. In stage 3 and 4 patients with EGFR mutation and undetected EGFR, survival in the LCF group was better than that in the single lung cancer group. Conversely, in stage 1 patients with mutant EGFR, the LCF group had worse survival than the single lung cancer group.

## 5. Conclusions and Limitations

The characteristics and outcomes of MPMs are important issues because the survival of patients with MPMs depends on baseline characteristics and treatments. The cumulative incidence of MPMs was 14.65% over a follow-up time of 14.7 years. The mean age at diagnosis of lung cancer in patients with LCF was lower than that in patients with single lung cancer. The overall mean survival in patients with LCF was better than that in patients with single lung cancer. Survival in patients with advanced lung cancer with mutant EGFR was superior to that of patients with undetected EGFR. However, this result was not observed in patients with early lung cancers.

The limitations of this study are common to studies utilizing large databases. Some coding may have been incomplete, especially regarding personal behavior, such as smoking and alcohol drinking. The results concerning survival in advanced stages of lung cancer with mutant EGFR compared with that in stage 1 with EGFR were notable. Data may have been missing because EGFR screening is not recommended for early-stage lung cancer. In our study of real-world practice, complete molecular information in all stages of lung cancers was not always provided, which is another limitation of this study. The study did not analyze ethnicity, and risk factors for MPMs were not discussed. Thus, further studies are required to clarify any discrepancy. In this study, we investigated patients with lung cancer in Taiwan. Similar to other Asian countries, EGFR mutations account for half of lung adenocarcinomas. This difference in ethnicity characteristics highlights the need for personalized treatment and follow-up in each country. Finally, we expect that the results of this study will lead to better personalized treatments and survival prediction along with reductions in the medical costs for treating MPMs.

## Figures and Tables

**Table 1:** Baseline characteristics of patients with multiple primary malignancies involving lung cancer.

Characteristics		Two or More Malignancies Involving Lung Cancer (*n* = 10,577)	
		LCF (*n* = 3728)	OCF (*n* = 6849)
		*n* (%)	*n* (%)
Age (years)	<65	1797 (48.20)	2390 (34.9)
	>65	1931 (51.8)	4459 (65.1)
Diagnostic age of first and second primary malignancies (mean [SD])	First primary malignancy	65.18 (11.86)	65.28 (12.09)
	Second primary malignancy	66.03 (11.96)	68.92 (12.08)
Median interval between the onset of the two primary malignancies (year)		0.11	3.26
Sex	Female	1599 (42.89)	2610 (38.11)
	Male	2129 (57.11)	4239 (61.89)
Hx of smoking	Yes	1233 (33.07)	2014 (29.41)
	No	1750 (46.94)	3893 (56.84)
	Unknown	745 (19.98)	942 (13.75)
Hx of alcohol drinking	Yes	602 (16.15)	1042 (15.21)
	No	2271 (60.92)	4749 (69.34)
	Unknown	855 (22.93)	1058 (15.45)
SMPM or MMPM	SMPM	2443 (65.53)	378 (5.52)
	MMPM	1285 (34.47)	6471 (94.48)
Stage of lung cancer	0	120 (3.22)	277 (4.04)
	1	1152 (30.9)	1653 (24.13)
	2	266 (7.14)	903 (13.18)
	3	498 (13.36)	1037 (15.14)
	4	1104 (29.61)	2161 (31.55)
	Unknown	588 (15.77)	818 (11.94)
Histological type of lung cancer	AC	2456 (65.88)	4149 (60.58)
	SCC	491 (13.17)	1513 (22.09)
	Small cell	395 (10.6)	605 (8.83)
	Others	386 (10.35)	582 (8.50)
EGFR	Mutant	638 (17.11)	755 (11.02)
	Not detected	480 (12.88)	649 (9.48)
	Unknown	2610 (70.01)	5445 (79.5)
Operation	Lobectomy	1750 (46.94)	2512 (36.68)
	Pneumonectomy	4 (0.11)	5 (0.07)
	No	1974 (52.95)	4332 (63.25)

AC: adenocarcinoma; EGFR, epidermal growth factor receptor; LCF, lung cancer first; MMPM: metachronous MPM; MPM: multiple primary malignancies; OCF, other cancer first; SCC: squamous cell carcinoma; SMPM: synchronous MPM.

**Table 2:** Characteristics of patients with single lung cancer and LCF.

Characteristics		Single Lung Cancer (*n* = 61,642)	LCF (*n* = 3728)	*p*
		*n* (%)	*n* (%)	
Mean diagnostic age of lung cancer (mean (SD))		67.12 (12.87)	65.18 (11.86)	<0.001
Age (years)	<65	26,163 (42.44)	1797 (48.20)	<0.001
	>65	35,479 (57.56)	1931 (51.8)	
Diagnostic age of first and second primary malignancies (mean [SD])	First primary malignancy	67.12 (12.87)	65.18 (11.86)	<0.001
	Second primary malignancy	-	66.03 (11.96)	-
Sex	Female	25,268 (40.99)	1599 (42.89)	0.022
	Male	36,374 (59.01)	2129 (57.11)	
Hx of smoking	Yes	24,121 (39.13)	1233 (33.07)	<0.001
	No	27,997 (45.42)	1750 (46.94)	
	Unknown	9524 (15.45)	745 (19.98)	
Hx of alcohol drinking	Yes	11,132 (18.06)	602 (16.15)	<0.001
	No	39,434 (63.97)	2271 (60.92)	
	Unknown	9524 (15.45)	855 (22.93)	
Time of occurrence	SMPM	-	0.02	-
	MMPM		2.35	
Stage of lung cancer	0	577 (0.94)	120 (3.22)	<0.001
	1	9974 (16.18)	1152 (30.9)	
	2	2588 (4.2)	266 (7.14)	
	3	8849 (14.36)	498 (13.36)	
	4	34,555 (56.06)	1104 (29.61)	
	Unknown	5099 (8.27)	588 (15.77)	
Histological type of lung cancer	AC	41,383 (67.13)	2456 (65.88)	<0.001
	SCC	9212 (14.94)	491 (13.17)	
	Small cell	6115 (9.92)	395 (10.6)	
	Others	4932 (8)	386 (10.35)	
EGFR	Mutant	15,287 (24.8)	638 (17.11)	<0.001
	Not detected	11,255 (18.26)	480 (12.88)	
	Unknown	35,100 (56.94)	2610 (70.01)	
Operation	Lobectomy	15,514 (25.17)	1750 (46.94)	<0.001
	Pneumonectomy	86 (0.14)	4 (0.11)	
	No	46,042 (74.69)	1974 (52.95)	

AC: adenocarcinoma; EGFR, epidermal growth factor receptor; Hx, history; LCF, lung cancer first; MMPM: metachronous MPM; MPM, multiple primary malignancies; MPM: multiple primary malignancies; SCC: squamous cell carcinoma.

**Table 3:** Mean survival time in patients with single lung cancer and LCF.

Characteristics		Single Lung Cancer (*n* = 61,642)	LCF (*n* = 3728)	*p*
**Mean (std)**				
Stage of lung cancer	0	4.33 (1.37)	4.37 (2.06)	0.8407
	1	4.82 (2.01)	4.76 (1.98)	0.3277
	2	3.50 (2.40)	4.07 (2.39)	0.0002
	3	2.22 (2.14)	2.96 (2.27)	<0.001
	4	1.35 (1.55)	1.80 (1.78)	<0.001
Hx of smoking	Yes	1.75 (2.01)	2.791 (2.33)	<0.001
	No	2.79 (2.32)	3.88 (2.32)	<0.001
	Unknown	1.73 (1.98)	2.42 (2.23)	<0.001
Hx of alcohol drinking	Yes	1.93 (2.12)	3.06 (2.43)	<0.001
	No	2.42 (2.27)	3.57 (2.37)	<0.001
	Unknown	1.73 (1.98)	2.42 (2.23)	<0.001
EGFR	Mutant	2.58 (1.96)	3.51 (1.97)	<0.001
	Not detected	1.77 (1.91)	2.78 (2.20)	<0.001
	Unknown	2.20 (2.37)	3.24 (2.50)	<0.001
Histological type of lung cancer	AC	2.63 (2.26)	3.77 (2.29)	<0.001
	SCC	1.56 (1.90)	2.57 (2.34)	<0.001
	Small cell	0.96 (1.35)	1.47 (1.67)	<0.001
	Others	1.56 (2.22)	2.46 (2.41)	<0.001

AC: adenocarcinoma; Hx, history; LCF, lung cancer first; SCC: squamous cell carcinoma.

**Table 4:** Most common second primary malignancies in patients with LCF.

Order	Most Common Second Primary Malignancies (ICD-9)	Total Population
		N (%)
1	Malignant neoplasm of bronchus and lung (162)	1866 (17.64)
2	Malignant neoplasm of hepatic flexure colon (153)	932 (8.81)
3	Malignant neoplasm of female breast, nipple, and areola (174)	841 (7.95)
4	Malignant neoplasm of prostate (185)	685 (6.48)
5	Malignant neoplasm of liver, primary (155)	659 (6.23)
6	Malignant neoplasm of the rectosigmoid junction (154)	597 (5.64)
7	Malignant neoplasm of skin of lip (173)	573 (5.42)
8	Malignant neoplasm of trigone of the urinary bladder (188)	458 (4.33)
9	Malignant neoplasm of the cardia of the stomach (151)	372 (3.52)
10	Malignant neoplasm of endocervix (180)	332 (3.14)

**Table 5:** Kaplan–Meier analysis of overall survival among different stages of patients with epidermal growth factor receptor (EGFR) mutations.

Stage of Lung Cancer			LCF							
			Stage 1	Stage 2	Stage 3	Stage 4	Stage 1	Stage 2	Stage 3	Stage 4
			EGFR+	EGFR+	EGFR+	EGFR+	EGFR−	EGFR−	EGFR−	EGFR−
Single lung cancer	Stage 1	EGFR+	<0.001							
	Stage 2	EGFR+		0.8602						
	Stage 3	EGFR+			0.7337					
	Stage 4	EGFR+				<0.001				
	Stage 1	EGFR−					0.6875			
	Stage 2	EGFR−						0.7051		
	Stage 3	EGFR−							0.0429	
	Stage 4	EGFR−								<0.001

## Data Availability

Not applicable.

## Supplementary Materials

The following supporting information can be downloaded at: https://www.mdpi.com/article/10.3390/jcm11195944/s1, Table S1: Mean survival time of single and second primary malignancy; Table S2: Univariate and multivariate regression analysis of overall survival among single lung cancer and lung cancer first patients.
