# Supplementary material for: Survival Analysis in Patients with Lung Cancer and Subsequent Primary Cancer: A Nationwide Cancer Registry Study
Source: J Clin Med. 2022 Oct 8;11(19):5944. doi: 10.3390/jcm11195944 (PMC9571412; doi:10.3390/jcm11195944)
Supplement: Supplementary file 1 [file jcm-11-05944-s001.zip › jcm-1933415-supplementary.pdf]

Table S1. Mean survival time of single and second primary malignancy.

Characteristics		Single lung cancer	LCF	P
**Mean, std**				
Stage of lung cancer	0	4.333(1.372)	4.373(2.061)	0.8407
	1	4.824(2.014)	4.762(1.985)	0.3277
	2	3.501(2.401)	4.077(2.391)	0.0002
	3	2.227(2.142)	2.965(2.274)	<0.001
	4	1.352(1.551)	1.802(1.780)	<0.001
Hx of smoking	Yes	1.751(2.012)	2.791(2.333)	<0.001
	No	2.790(2.326)	3.888(2.322)	<0.001
	Unknown	1.738(1.987)	2.427(2.234)	<0.001
Hx of alcohol drinking	Yes	1.937(2.129)	3.064(2.432)	<0.001
	No	2.426(2.277)	3.573(2.371)	<0.001
	Unknown	1.738(1.987)	2.427(2.234)	<0.001
Histological type of lung cancer	Adenocarcinoma	2.631(2.261)	3.770(2.293)	<0.001
	Squamous cell carcinoma	1.562(1.909)	2.572(2.345)	<0.001
	Small cell	0.965(1.355)	1.475(1.675)	<0.001
	Others	1.564(2.223)	2.461(2.416)	<0.001

AC: Adenocarcinoma; SCC: Squamous cell carcinoma.

Table S2. Univariate and multivariate regression analysis of overall survival among single lung cancer and lung cancer first patients.

Characteristics		Total Population		Univariate analysis		Multivariate analysis	
		**N**	**%**	HR (95%CI)	P	HR (95%CI)	P
Group	LCF	3728	5.70	1	<0.001	1	<0.001
	Single Lung	61642	94.30	1.60(1.54-1.67)		1.19(1.14-1.24)	
Age	<65	27960	42.77	1	<0.001	1	<0.001
	>65	37410	57.23	1.91(1.88-1.95)		1.64(1.61-1.67)	
Gender	Female	26867	41.10	1	<0.001	1	<0.001
	Male	38503	58.90	1.74(1.71-1.77)		1.29(1.26-1.32)	
Hx of smoking	No	29747	45.51	1	<0.001	1	<0.001
	Yes	25354	38.79	1.82(1.78-1.85)		1.13(1.10-1.16)	
	Unknown	10269	15.71	1.91(1.86-1.96)		1.38(1.30-1.46)	
Hx of alcohol drinking	No	41705	63.80	1	<0.001	1	<0.001
	Yes	11734	17.95	1.34(1.31-1.37)		1.00 (0.98-1.03)	
	Unknown	10379	15.88	1.51(1.48-1.55)		0.89(0.85-0.94)	
Stage of lung cancer	1	11126	17.02	1	<0.001	1	<0.001
	0	697	1.07	0.41 (0.31-0.53)		0.52(0.40-0.68)	
	2	2854	4.37	3.56(3.34-3.79)		2.26(2.12-2.41)	
	3	9347	14.40	7.16 (6.84-7.51)		3.34(3.17-3.51)	
	4	35659	54.55	12.24(11.73-12.77)		6.39(6.09-6.72)	
	Unknown	5687	8.70	7.74(7.36-8.14)		3.36(3.18-3.56)	
Histological type of lung cancer	EAC	43839	67.06	1	<0.001	1	<0.001
	SCC	9703	14.84	1.88(1.83-1.92)		1.32(1.28-1.36)	
	Small cell	6510	9,96	2.85(2.77-2.93)		1.49(1.45-1.54)	
	Others	5318	8.14	1.93(1.87-1.99)		1.51(1.46-1.57)	
EGFR	Non-detected	11735	17.95	1	<0.001	1	<0.001
	Mutant	15925	24.36	0.67(0.65-0.68)		0.62(0.60-0.64)	
	Unknown	37710	57.69	0.81(0.79-0.83)		0.96(0.93-0.99)	
Operation	No	48016	73.45	1	<0.001	1	<0.001
	Lobectomy	17264	26.41	0.14(0.13-0.14)		0.36(0.35-0.38)	
	Pneumonectomy	90	0.14	0.28(0.22-0.38)		0.47(0.36-0.62)	

AC: Adenocarcinoma; SCC: Squamous cell carcinoma.
